# Russian–United States vaccine science diplomacy: Preserving the legacy

**DOI:** 10.1371/journal.pntd.0005320

**Published:** 2017-05-25

**Authors:** Peter J. Hotez

**Affiliations:** 1 Texas Children’s Hospital Center for Vaccine Development, Departments of Pediatrics and Molecular Virology and Microbiology, National School of Tropical Medicine, Baylor College of Medicine, Houston, Texas, United States of America; 2 James A Baker III Institute for Public Policy, Rice University, Houston, Texas, United States of America; 3 Department of Biology, Baylor University, Waco, Texas, United States of America; 4 Scowcroft Institute of International Affairs, Bush School of Government and Public Service, Texas A&M University, College Station, Texas, United States of America; National Institutes of Health, UNITED STATES

United States–Russia tensions over the hostilities in Ukraine, collapsed cease-fires and chemical weapons use in Syria, and accusations of alleged cyberattacks may require a diplomatic reset. To help ease growing strains and restore dialogue and cooperation, it is worth looking to a productive and extraordinary historical record of international scientific collaborations.

Throughout the last half of the 20th century, the United States and Soviet Union managed a complex Cold War foreign policy relationship by opening and maintaining channels in sports, the arts, literature, and other humanitarian endeavors. One of the most productive engagements was through a mostly clandestine joint initiative to develop, test, and deliver life-saving vaccines that targeted the ancient scourges of humankind. Ultimately, through Cold War vaccine diplomacy, smallpox was eradicated, and polio was mostly eliminated [1].

In 1956, a year before the Sputnik launch, the US State Department and its counterpart in the Soviet Union facilitated links between the American virologist Dr. Albert Sabin and two Soviet virologists, Drs. Mikhail Chumakov and Anatoli Smorodintsev, to collaborate in producing the oral polio vaccine at a scale suitable for testing it first on millions of Soviet citizens [2]. Watched closely by a suspected KGB operative, the Russian virologists visited Sabin in his Cincinnati Children’s Hospital research laboratory, and this was followed by Sabin’s reciprocal visit to Moscow in the same year [2]. Within two years, a shipment of Sabin’s polio virus strains arrived in the Soviet Union on dry ice.

The vaccine was scaled up and produced in Chumakov’s laboratory, but clinical testing required him to bypass an obstructionist Soviet Minister of Health and go directly to the Kremlin leadership for large-scale trials to proceed [2]. Through the Sabin–Chumakov collaboration, the vaccine was tested initially on millions of school children and subsequently on young adults. A World Health Organization (WHO) representative confirmed the safety of the trials and the vaccine’s ability to prevent poliomyelitis. The vaccine has now been used to stop polio transmission everywhere except Afghanistan and Pakistan.

On the heels of the polio vaccine licensure, Soviet scientists developed a unique process for preserving the smallpox vaccine in harsh environments to produce hundreds of millions of doses of freeze-dried vaccine. This proved to be a key enabling technology for the American public health physician D. A. Henderson (who passed away in 2016) to lead a celebrated campaign that eradicated smallpox by 1977 [1]. Indeed, it was Viktor Zhdanov, a Soviet virologist and deputy health minister, who (in 1958) first proposed the concept of smallpox eradication to the WHO and led vaccine production and donation efforts [3].

Throughout the late 20th century, joint US–Soviet or US–Russian health activities continued, with a major focus on HIV/AIDS prevention, as well as the prevention of other sexually transmitted diseases and tuberculosis (TB) [4]. Then, in 2009, a Bilateral Presidential Commission (BPC) between the US and Russia was established, which included joint cooperation in the areas of polio eradication, malaria control, studies on noncommunicable diseases (NCDs) related to alcohol and tobacco consumption, and expanding the use of mobile phone technology for maternal health care [4]. The BPC was subsequently strengthened in 2011 through a Carnegie Endowment for International Peace public–private task force [4].

However, these important efforts still fall short of the compelling stories offered by the joint vaccine science diplomacy that led to the oral polio vaccine now leading to global eradication efforts. Could Cold War lessons in this arena ease today’s escalating tensions between the US and Russian governments? Hostilities between the United States and Russia may be nowhere near our confrontations during the 1950s, 1960s, and 1970s, but extraordinary opportunities remain to meld our scientific activities to eliminate the world’s major neglected and emerging diseases (Fig 1).

**Fig 1:**
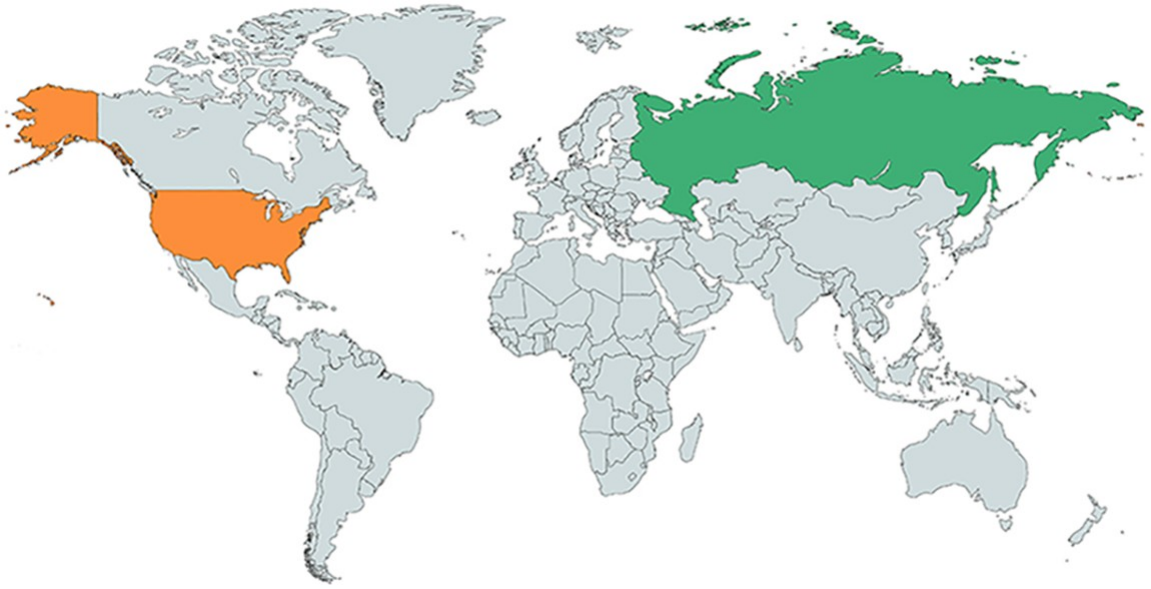
Russia–United States. Original figure created with mapchart.net (https://mapchart.net/).

Within the last five years, we have seen the rise or emergence of several catastrophic infectious diseases for which we have no vaccine. They include Middle East Respiratory Syndrome (MERS), Ebola, and Zika virus infections, just to name a few. To help provide global financing schemes for such a vaccine, a new Coalition for Epidemic Preparedness Innovations (CEPI) is being established as an international funding organization akin to the Global Fund to Fight AIDS, Tuberculosis and Malaria [5]. Russia is at special risk for many of these infectious diseases, which often arise as outbreaks in conflict or postconflict settings due to the collapse of health care infrastructures. For example, infectious diseases such as measles and polio have re-emerged in the war zones of Syria and Libya, and hundreds of thousands of cases of a neglected tropical disease known as leishmaniasis are now occurring in these areas of the Middle East, as well as in Afghanistan, where they are also affecting US troops [6, 7]. There are concerns that such infections could spread to the Russian Federation. At the same time, new reports indicate a similar emergence of leishmaniasis in Dagestan, Russia’s southernmost republic and a region beset by conflict from neighboring Chechnya, as well as in eastern Crimea [8].

In addition to regional conflicts, another factor promoting the rise of neglected tropical infections is poverty. Previously, I pointed out the rise of poverty in selected areas of Russia [9]. According to the World Bank, the Russian economy is in recession, with almost 15% of the Russian population (or almost 20 million people) now living in poverty [9]. Economic recession is in part due to decreases in oil prices (that represent Russia’s leading export), sanctions, and decreased investments [9, 10].

As a consequence of poverty, possibly together with other factors such as climate change and human migrations now also affecting southern Europe [11], we may be seeing a rise in poverty-related neglected diseases. In addition to leishmaniasis, other neglected tropical diseases now on the rise include vivax malaria [12], West Nile virus (WNV) and other arbovirus infections [13], and helminthic zoonoses such as opisthorchiasis and echinococcosis [14, 15]. Russia also has high rates of TB, including multi-drug-resistant (MDR) TB [16], which likely combines with the nation’s high rates of NCDs to produce co-morbidities. The nonprofit organization Partners in Health (PIH) is actively pursuing MDR TB control efforts in Russia [17], as is Medicins Sans Frontiers (MSF) [18].

It is feasible to develop and test innovative vaccines for diseases such as leishmaniasis, WNV, TB, and *Plasmodium vivax*-caused malaria if both countries committed scientific effort and resources to make it happen. A joint US–Russian initiative to develop new neglected disease vaccines is an achievable goal and one that could begin through the nonprofit Sabin Vaccine Institute, which has already launched similar US-led initiatives with Muslim-majority countries in the Middle East and North Africa, as well as Malaysia [6].

Since the 1950s, joint US–Russian cooperation has shown a dual track record of improving both foreign relations and scientific collaborations. Vaccine science diplomacy is not a panacea for heightened tensions between the US and Russia, but the approach has proven valuable for promoting joint humanitarian efforts while simultaneously producing life-saving vaccines.

## Supporting information

S1 File Russian translation of editorial pntd.0005320, " Russian–United States vaccine science diplomacy: Preserving the legacy." (PDF)
