# Supplementary material for: Russian–United States vaccine science diplomacy: Preserving the legacy
Source: PLoS Negl Trop Dis. 2017 May 25;11(5):e0005320. doi: 10.1371/journal.pntd.0005320 (PMC5444589; doi:10.1371/journal.pntd.0005320)
Supplement: S1 File — (PDF) [file pntd.0005320.s001.pdf]

Редакционная статья PLOS NTDs

Научная «вакцинная дипломатия» между Россией и США: сохраним наследие

Питер Дж. Хотез (Peter J Hotez)[1,2,3,4]

[1]Институт вакцины им. Сэбина и Центр разработки вакцины детской больницы Техаса, отделения педиатрии, молекулярной вирусологии и микробиологии, Национальная школа тропической медицины, Бейлорский медицинский колледж, Хьюстон, Техас, США

[2]Институт общественной политики имени Джеймса Бейкера III, Университет Райса, Хьюстон, Техас, США

[3]Факультет биологии, Университет Бейлор, Уэйко, Техас, США

[4]Институт международных отношений Скоукрофта, Школа управления и государственной службы имени Буша, Техасский университет A&M, Колледж-Стейшн, Техас, США

Статья содержит 1151 слово, список литературы из 18 источников, а также 1 рисунок.

Чтобы снизить напряжение в американско-российских отношениях, возросшее из-за военных действий в Украине, прекращения перемирия в Сирии и подозрений кибератаках, может потребоваться дипломатическая «перезагрузка». Для восстановления продуктивного диалога и сотрудничества стоит воспользоваться богатым опытом совместной реализации международных научных проектов.

Во второй половине 20 века США и Советский Союз решили глобальную проблему «холодной войны», проводя совместные кампании в сфере спорта, искусства, литературы и других гуманитарных видов деятельности. Одним из наиболее эффективных проектов стала не предававшаяся широкой огласке инициатива, направленная на разработку, тестирование и выпуск жизненно важных вакцин против болезней, издавна поражавших человечество. В конечном итоге, именно вследствие «вакцинной дипломатии» периода холодной войны была побеждена натуральная оспа и практически уничтожен полиомиелит [1].

В 1956 году, за год до запуска первого спутника Земли, Государственный департамент США и соответствующее учреждение в СССР способствовали установлению отношений между американским вирусологом доктором Альбертом Сэбином и двумя советскими вирусологами – докторами Михаилом Чумаковым и Анатолием Смородинцевым. Они совместно разработали пероральную полиомиелитную вакцину, масштаб производства которой позволил протестировать ее на миллионах советских граждан [2]. Поездка советских вирусологов к доктору Сэбину в исследовательскую лабораторию детской больницы Цинциннати проходила под пристальным вниманием сотрудников КГБ, и в том же году Сэбин нанес ответный визит в Москву [2]. В течение двух лет в СССР поставлялись штаммы полиовируса Сэбина в упаковках с сухим льдом.

Вакцина разрабатывалась и производилась в лаборатории Чумакова, но за разрешением на проведение масштабных клинических испытаний ему пришлось в обход недоброжелательно настроенного министра здравоохранения обратиться непосредственно к кремлевскому руководству [2]. Препарат, разработанный Сэбином и Чумаковым, протестировали на миллионах школьников, а затем на молодых людях. Представитель Всемирной организации здравоохранения (ВОЗ) подтвердил эффективность опытных образцов и способность вакцины предотвратить полиомиелит. После этого вакцину стали использовать для борьбы с полиомиелитом во всех странах, кроме Афганистана и Пакистана.

После получения разрешения на использование полиовакцины советские ученые разработали уникальную методику хранения вакцины против оспы в агрессивной среде, что позволило произвести сотни миллионов доз лиофилизированной вакцины. Благодаря использованию этой методики работавший в системе общественного здравоохранения американский врач Д. Хендерсон (скончался в 2016 году) инициировал знаменитую кампанию по ликвидации оспы, увенчавшуюся успехом в 1977 году [1]. На самом деле инициатором Программы глобальной ликвидации оспы был Виктор Жданов, советский вирусолог и заместитель министра здравоохранения, который в 1958 году впервые предложил ВОЗ концепцию ликвидации оспы и содействовал производству вакцины, а также процессу вакцинации [3].

В конце двадцатого века совместные американо-советские и американо-российские мероприятия в области здравоохранения были нацелены на профилактику ВИЧ/СПИДа и других заболеваний, передающихся половым путем, а также туберкулеза (ТБ) [4]. В 2009 году была создана Российско-американская двусторонняя Президентская комиссия, деятельность которой включала сотрудничество в сфере ликвидации полиомиелита, борьбы с малярией, проведение исследований по неинфекционным заболеваниям, связанным с потреблением алкоголя и табака, а также расширение использования технологии мобильной связи для охраны здоровья матери [4]. Позже, в 2011 году, Президентской комиссии оказал существенную поддержку Фонд Карнеги за международный мир [4].

Однако эти усилия не достигли бы цели, если бы не были подкреплены убедительными примерами научного сотрудничества, благодаря которому была создана пероральная полиомиелитная вакцина, способствовавшая ликвидации очагов заболевания по всему миру. Могут ли примеры сотрудничества в сфере здравоохранения в эпоху холодной войны помочь наладить взаимопонимание между правительствами США и России? Противостояние между США и Российской Федерацией напоминает о конфронтации 50-х, 60-х и 70-х годов 20 века, и следует принять чрезвычайные меры для объединения научного потенциала обеих стран с целью уничтожения опаснейших ранее забытых и новых распространившихся по миру заболеваний (рис. 1).

Рис. 1. Россия — США. Рисунок создан с использованием ресурса mapchart.net: https://mapchart.net/.

В течение последних пяти лет отмечается появление и распространение нескольких опаснейших инфекционных заболеваний, от которых не существует вакцины. К ним, в частности, относятся вирус MERS (ближневосточный респираторный коронавирусный синдром), лихорадка Эбола и вирус Зика. С целью создания схем международного финансирования разработки и производства вакцин от этих вирусов была создана Коалиция по инновациям против пандемических угроз. Механизм ее финансирования похож на использующийся для Глобального фонда борьбы со СПИДом, туберкулезом и малярией [5]. В отношении многих из этих инфекционных заболеваний Россия находится в зоне риска, который возрастает для стран с военными конфликтами, поскольку в них часто страдает инфраструктура системы здравоохранения. Например, в зонах военных действий в Сирии и Ливии вновь распространяются такие инфекционные заболевания, как корь и полиомиелит. Кроме того, в этих регионах Ближнего Востока и в Афганистане зафиксированы сотни тысяч случаев заболевания такой, казалось бы, забытой тропической болезнью, как лейшманиоз, в том числе и среди и американского военного контингента [6, 7]. Есть опасения, что эти инфекции могут распространиться и в Российской Федерации. По последним данным зафиксировано появление лейшманиоза в Дагестане, самой южной республике России, соседствующей с Чечней, где происходил вооруженный конфликт, а также в восточной части Крыма [8].

Распространению тропических заболеваний, о которых уже забыли, способствуют не только региональные конфликты, но и бедность. Ранее я указывал на рост бедности в некоторых регионах России [8]. Согласно данным Всемирного банка, экономика России пребывает в упадке. На сегодняшний день 13,3 % населения страны, или практически 20 миллионов людей, живут за чертой бедности [9]. Спад экономики частично происходит по причине снижения цен на нефть (главная статья экспорта в России), санкций и сокращения инвестиций [9, 10].

Вследствие роста бедности, возможно, в сочетании с другими факторами, такими как климатические изменения и миграция населения, которые затрагивают и Южную Европу [11], может начаться распространение связанных с бедностью болезней, которым не уделяют должного внимания. Кроме лейшманиоза, в списке тропических заболеваний, которым не уделяют должного внимания, сегодня находятся трехдневная малярия [12], лихорадка Западного Нила (WNV) и другие арбовирусные инфекции [13], такие как описторхоз и эхинококкоз [14, 15]. Кроме того, в России очень высок уровень заболеваемости туберкулезом, в частности мультирезистентной формой [16], при которой повышается вероятность возникновения сопутствующих патологий. В особенности в сочетании с неинфекционными заболеваниями, которые также весьма интенсивно распространяются. Некоммерческие организации Partners in Health (PIH) и Médecins Sans Frontières (MSF) активно принимают меры по контролю за распространением мультирезистентной формы туберкулеза в России [17, 18].

Разработать и протестировать инновационные вакцины от таких болезней, как лейшманиоз, WNV, туберкулез и трехдневная малярия, вполне по силам. Однако для этого в обеих странах должна быть проведена соответствующая научная работа и обеспечены необходимые ресурсы. Совместная американско-российская разработка новых вакцин против заболеваний, которым не уделяют должного внимания, — цель вполне достижимая. Начало может быть положено благодаря усилиям некоммерческой организации Институт вакцины Сэбина (Sabin Vaccine Institute), которая уже начала реализацию подобных инициатив в мусульманских странах на Ближнем Востоке, в Северной Африке и Малайзии [6].

Начиная с 1950-х годов кооперация США и России дает неизменно высокие результаты в сфере международных отношений и научного сотрудничества. Научная «вакцинная дипломатия» — это, конечно, не панацея от обострения напряженности между США и Россией. Однако она вносит существенный вклад в развитие совместной гуманитарной деятельности и производство жизненно необходимых вакцин.

Источники

1. Hotez PJ (2014) “Vaccine Diplomacy”: Historical Perspectives and Future Directions («Вакцинная дипломатия»: исторические аспекты и перспективы). PLoS Negl Trop Dis 8(6): e2808.
2. Swanson W (2012) Birth of a cold war vaccine (Создание вакцины во время холодной войны). Sci Am 306(4): 66-9.
3. Henderson DA (2009) Smallpox: The Death of a Disease (Оспа: победа в борьбе с болезнью), Prometheus Books, pp. 61-2.
4. Rojansky M, Tabarovsky I (2013) The latent power of health cooperation in US-Russian relations (Скрытая сила сотрудничества между США и Россией в области здравоохранения). Science & Diplomacy (Наука и дипломатия). Июнь. http://www.sciencediplomacy.org/article/2013/latent-power
5. CEPI. http://cepi.net/. Опубликовано 14 января 2017 г.
6. Hotez PJ (2015) Vaccine Science Diplomacy: Expanding Capacity to Prevent Emerging and Neglected Tropical Diseases Arising from Islamic State (IS)–Held Territories (Вакцинная дипломатия: план действий по предотвращению распространения новых и забытых тропических болезней, возникающих на территории исламских государств). PLoS Negl Trop Dis 9(9): e0003852.
7. Beaumier CM, Gomez-Rubio AM, Hotez PJ, Weina PJ (2013) United States Military Tropical Medicine: Extraordinary Legacy, Uncertain Future (Военная тропическая медицина США: пережитки прошлого, неопределенность будущего). PLoS Negl Trop Dis 7(12): e2448.
8. Понировский Е. Н., Стрелкова М. В., Завойкин В. Д., Тумольская Н. И., Мазманян М. В. и др. (2015 г.) [ЭПИДЕМИОЛОГИЧЕСКАЯ СИТУАЦИЯ ПО ЛЕЙШМАНИОЗАМ В РОССИЙСКОЙ ФЕДЕРАЦИИ: ПЕРВЫЕ ДОСТОВЕРНЫЕ СЛУЧАИ МЕСТНОЙ ПЕРЕДАЧИ]. Медицинская паразитология и паразитарные болезни (Москва). 2015 г., июл-сен;(3):3-7.
9. Hotez PJ (2016) Blue Marble Health: An Innovative Plan to Fight Diseases of the Poor amid Wealth (Здоровье планеты: инновационный план по борьбе с болезнями бедности среди богатства). Johns Hopkins University Press; p. 119.
10. Всемирный банк. http://www.worldbank.org/en/country/russia. Опубликовано 7 ноября 2016 г.
11. Hotez PJ (2016) Southern Europe’s Coming Plagues: Vector-Borne Neglected Tropical Diseases (Грядущие эпидемии Южной Европы: забытые трансмиссивные тропические болезни). PLoS Negl Trop Dis 10(6): e0004243.
12. Баранова А. М., Ежов М. Н., Гузеева Т. М., Морозова Л. Ф. (2013 г.), Современная маляриологическая ситуация в странах СНГ (2011–12 гг.). Медицинская паразитология и паразитарные болезни (Москва) (4): 7-10.
13. Сергиев В. П., Ганушкина Л. А., Филатов Н.Н. (2011 г.) [Новые и возвращающиеся переносчики вирусных лихорадок — угроза эпидемических осложнений на юге Европы и России]. Журнал микробиологии, эпидемиологии и иммунобиологии. 2011 г., июль-август; (4): 97-100.

14. Ogorodova LM, Fedorova OS, Sripa B, Mordvinov VA, Katokhin AV, et al. (2015) Opisthorchiasis: An Overlooked Danger (Описторхоз: упущенная из виду опасность). PLoS Negl Trop Dis 9(4): e0003563.
15. Konyaev SV, Yanagida T, Nakao M, Ingovatova GM, Shoykhet YN, et al. (2013) Genetic diversity of Echinococcus spp. in Russia. (Генетическое разнообразие видов эхинококка в России.) Parasitology (Паразитология) 140(13):1637-47.
16. Mokrousov I, Vyazovaya A, Solovieva N, Sunchalina T, Markelov Y, et al. (2015) Trends in molecular epidemiology of drug-resistant tuberculosis in Republic of Karelia, Russian Federation. (Тенденции молекулярной эпидемиологии туберкулеза с лекарственной устойчивостью в республике Корея, Российская Федерация) BMC Microbiology (Микробиология BMC) 15: 279.
17. Partners in Health. Россия. www.pih.org/country/russia. Опубликовано 7 ноября 2016 г.
18. Врачи без границ. http://www.doctorswithoutborders.org/country-region/russian-federation. Опубликовано 7 ноября 2016 г.
